# Poor Sleep in Multiple Sclerosis Correlates with Beck Depression Inventory Values, but Not with Polysomnographic Data

**DOI:** 10.1155/2016/8378423

**Published:** 2016-01-18

**Authors:** Christian Veauthier, Gunnar Gaede, Helena Radbruch, Klaus-Dieter Wernecke, Friedemann Paul

**Affiliations:** ^1^Interdisciplinary Center of Sleep Medicine, Charité University Medicine Berlin, Charitéplatz 1, 10117 Berlin, Germany; ^2^NeuroCure Clinical Research Center, Charité University Medicine Berlin, Charitéplatz 1, 10117 Berlin, Germany; ^3^Department of Neurology, St. Joseph Hospital Berlin-Weissensee, 13088 Berlin, Germany; ^4^CRO SOSTANA GmbH and Charité University Medicine Berlin, Wildensteiner Straße 27, 10318 Berlin, Germany; ^5^Clinical and Experimental Multiple Sclerosis Research Center, Department of Neurology, Charité University Medicine Berlin, 10117 Berlin, Germany; ^6^Experimental and Clinical Research Center, Max Delbrück Center for Molecular Medicine and Charité University Medicine Berlin, 13125 Berlin, Germany

## Abstract

*Objectives*. Pittsburgh Sleep Quality Index (PSQI) values correlate with depression, but studies investigating the relationship between PSQI values and polysomnographic (PSG) data showed inconsistent findings.* Methods*. Sixty-five consecutive patients with multiple sclerosis (MS) were retrospectively classified as “good sleepers” (GS) (PSQI ≤ 5) and “poor sleepers” (PS) (PSQI > 5). The PSG data and the values of the Visual Analog Scale (VAS) of fatigue, Modified Fatigue Impact Scale (MFIS), Fatigue Severity Scale (FSS), Epworth Sleepiness Scale (ESS), and the Beck Depression Inventory (BDI) were compared.* Results*. No significant differences were found either for PSG data or for ESS, MFIS, and FSS values; but PS showed significantly increased BDI and VAS values.* Conclusions*. Poor sleep is associated with increased depression and fatigue scale values.

## 1. Introduction

In several studies Pittsburgh Sleep Quality Index (PSQI) values [1] correlated with sleep diary variables and depression scales [2, 3], but not with actigraphic and polysomnographic (PSG) data [4–7].

The PSQI is a self-rated questionnaire, which assesses sleep quality and disturbances by seven component scores (subjective sleep quality, sleep latency, sleep duration, habitual sleep efficiency, sleep disturbances, use of sleeping medication, and daytime dysfunction). Good sleepers (GS) show PSQI values ≤ 5 whereas poor sleepers (PS) are defined by values > 5.

Poor sleep measured with the PSQI correlates with the diagnostic criteria of insomnia of the* Diagnostic and Statistical Manual of Mental Disorders (DSM) Fourth Edition* [8]. The PSQI is widely used as a screening instrument for insomnia [9].

In contrast to insomnia OSA is normally not associated with an increased sleep latency [10]. Macey et al. [7] did not find any relationship between PSQI values and OSA in 49 patients. Veauthier [11] classified 231 untreated OSA patients, 102 treated OSA patients, 22 insomnia patients, and 19 patients with restless legs syndrome (RLS) or periodic limb movement disorder (PLMD) in GS and PS. Whereas all insomnia patients, the majority of RLS/PLMD patients, and most untreated OSA patients were PS, almost half of the treated OSA patients were PS. In this study, untreated OSA GS had significantly more deep sleep compared with untreated OSA PS. The discrepancy of these two studies [10, 11] could be explained by the bigger sample size in the study by Veauthier and by the different study design: Macey et al. investigated the linear relationship between PSQI values and AHI, whereas Veauthier classified his patients into GS and PS and then analyzed the differences between the two subgroups.

Manzar et al. [12] found a* negative* correlation between sleep onset latency (SOL) and PSQI values in healthy students. All other PSG data did not show any difference.

Sleep disorders are frequent in MS. The aim of this retrospective study was to classify MS patients in GS and PS and to investigate whether poor sleep is associated with depression scores or with PSG data.

## 2. Methods

### 2.1. Participants

This is a retrospective analysis of the PSQI values from a previously published study investigating MS patients by home-based polysomnography (in the original work, the PSQI values have not been published) [13]. In the original study 66 MS patients (aged 20–66 years) were included from whom 49 patients were suffering from sleep disorders (OSA, *n* = 8; insomnia, *n* = 17; RLS/PLMD, *n* = 24). For more demographic details and performance and classification of the PSG please see the original article [13].

### 2.2. Measures and Procedures

In this retrospective data analysis, the following values were obtained.

#### 2.2.1. Polysomnographic Data

They include sleep efficiency (SE) (sleep in percent of time in bed, %/TiB), SOL in minutes, non-REM sleep 1 and non-REM sleep 2 (%/TiB), deep sleep (%/TiB), REM sleep (%/TiB), arousal-index (per hour sleep, /h), wake after sleep onset in minutes, number of sleep stage changes per night, awakenings per night, and periodic limb movement index (/h). Due to the small sample size of OSA patients the AHI and further respiratory parameters were not included in this analysis.

#### 2.2.2. Questionnaires

The questionnaires include PSQI [1], Visual Analog Scale (VAS) of fatigue scored 0–10 cm [14], Modified Fatigue Impact Scale (MFIS) [15], Fatigue Severity Scale (FSS) [14], Epworth Sleepiness Scale (ESS) [16], and the Beck Depression Inventory (BDI) [17].

### 2.3. Statistical Analysis

Patients were classified according to their PSQI values in GS (PSQI ≤ 5) and PS (PSQI > 5). Following an exploratory analysis of the data the Mann-Whitney* U* test for pairwise comparisons was performed. Statistical significance was established at <0.05. Due to the exploratory nature of the study, all tests were performed as exploratory data analyses, such that no adjustments for multiple testing have been made. Analysis was performed with SPSS software (IBM SPSS Statistics, Version 21, Copyright 1989, 2010 SPSS Inc., an IBM Company, Chicago, IL, USA).

## 3. Results

Table 1 displays the results. Forty-two patients were classified into PS and 23 into GS (from one patient no PSQI data were obtained). PSG data were not significantly different between the two subgroups.

The comparison of the questionnaires showed significant differences for the VAS of fatigue (PS: mean 5.6/SD ± 2.6; GS: mean 3.0/SD ± 2.3; *P* = 0.001) and BDI (PS: mean 12.9/SD ± 8.6; GS: mean 9.3/SD ± 8.2; *P* = 0.049), whereas the other questionnaires did not show any difference. In particular, the MFIS values and FSS values were higher in PS compared to GS but without statistical significance (mean MFIS values 40.9 versus 29.9; *P* = 0.053/mean FSS-values 5.0 versus 4.1; *P* = 0.184).

Twenty-nine patients (21 PS and 8 GS) were suffering from excessive daytime sleepiness (EDS) (according to ESS values ≥ 10). Although the ESS values were higher in PS, this was not significant (PS: mean 9.6/SD + 5.0; GS: mean 7.7/SD + 4.0; *P* = 0.112) (see Table 1).

## 4. Discussion

PS showed increased depression and fatigue scores compared to GS, whereas the PSG data were not significantly different between the two subgroups. These findings are in line with previous studies demonstrating a close relationship between PSQI values and depression whereas no relationship with PSG data could be found.

In insomnia, sleep disturbances are the key symptom of the disorder and the PSQI shows a sensitivity of almost 100 percent for insomnia [9, 11]. The PSQI is a good diagnostic screening tool for insomnia.

In the abovementioned study by Veauthier [11], however, only 72 percent of untreated OSA patients were PS. OSA can even be asymptomatic in moderate or severe OSA (high AHI without subjective sleep disturbances). In summary, in MS patients the PSQI alone without other questionnaires is not a reliable screening tool for sleep disorders in general (e.g., OSA).

Although the PS showed higher mean ESS values compared to GS, this difference was not significant. The mean ESS values were higher in PS as well as in GS compared to the ESS values of healthy controls in the literature [18]. Almost half of PS were suffering from EDS and almost one-third of GS. Sleep disorders were not significantly associated with ESS values but with fatigue [13]. The most frequent sleep disorders in the present study (insomnia, PLMD, and RLS) cause more likely fatigue than sleepiness [19]. Moreover, sleepiness is usually measured by multiple sleep latency tests (MSLT) and several studies showed inconsistent findings between the results of the ESS and the objective measurements by the MSLT, meaning that the ESS and the MSLT do not measure the same aspects of sleepiness [20]. In our study no MSLT was performed; therefore, we cannot compare the BDI values with objective measurements of sleepiness. Beyond that, associations between depressive symptoms and hypersomnia are complex and often bidirectional [21]. Of the many disorders associated with excessive sleepiness in the general population, the most frequent are mental health disorders, particularly depression [21].

The question remains, why PS show significantly increased BDI values. On the one hand, sleep disorders can lead to fatigue and there is an overlap between fatigue and depression [22]. Moreover, the prevalence of depression is increased in patients suffering from OSA or insomnia or RLS, and sleep disorders and depression are interdependent and influence each other [21, 23–25]. The fact that in the present study the BDI values were increased in PS could be due to depression itself or it might be the consequence of an underlying sleep disorder. An overestimation of sleep problems is possible as well: in the study by McCrae et al. [26] noncomplaining GS had poorer objective sleep quantity than complaining PS. Moreover, insomnia patients underestimate their SE whereas healthy persons overestimate their SE [27]. However, MS patients with increased BDI values (whether suffering from clinically defined depression or not) tend to perceive their sleep as disturbed and almost two-thirds of consecutive MS patients report poor sleep quality in this study. This presents a challenge for the treatment of MS patients and further studies are needed in regard to this issue.

## 5. Limitations

This is a retrospective data analysis with a limited sample size; further prospective studies are needed.

## 6. Conclusions

In this retrospective study of MS patients, PS showed increased depression and fatigue scale values compared to GS, whereas PSG data were not significantly different between the two subgroups.

## Figures and Tables

**Table 1:** Demographic data and results of questionnaires and polysomnography.

		All	Poor sleepers	Good sleepers	*P* values
	*n* (w/m)	65 (44/21)	42 (29/13)	23 (15/8)	*0.787*
Age (years)	Mean ± SD	44.6 ± 10.0	46.4 ± 10.4	41.5 ± 8.7	*0.051*
	25%	39.8	40.8	32.0	
	75%	50.0	52.3	48.0	
Disease duration (years)	Mean ± SD	12.4 ± 9.0	13.4 ± 10.1	10.2 ± 6.3	*0.562*
	25%	5.0	5.0	4.8	
	75%	18.5	20.8	13.0	
Sleep disorder	Insomnia	17	15	2	*0.012*
	OSA	8	5	3	
	PLMD/RLS	23	16	7	
	None	17	6	11	

VAS	Mean ± SD	4.7 ± 2.7	5.6 ± 2.6	3.0 ± 2.3	***0.001***
	25%	2.8	4.2	1.1	
	75%	7.0	7.3	4.3	
MFIS	Mean ± SD	37 ± 20.4	40.9 ± 18.3	29.9 ± 22.8	*0.053*
	25%	20.8	31.8	18.0	
	75%	53.0	53.8	52.0	
PSQI	Mean ± SD	7.7 ± 4.2	10.0 ± 3.5	3.6 ± 1.4	***<0.0001***
	25%	5.0	7.0	2.0	
	75%	11.0	13.0	5.0	
ESS	Mean ± SD	8.9 ± 4.7	9.6 ± 5.0	7.7 ± 4.0	*0.112*
	25%	5.3	6.3	3.0	
	75%	13.0	14.0	10.0	
BDI	Mean ± SD	11.7 ± 8.6	12.9 ± 8.6	9.3 ± 8.2	***0.049***
	25%	5.0	12.0	7.0	
	75%	17.3	18.3	12.0	
FSS	Mean ± SD	4.6 ± 2.2	5.0 ± 2.3	4.1 ± 2.0	*0.184*
	25%	3.1	3.8	2.5	
	75%	6.4	6.4	6.0	

Arousal-index (*n*/h)	Mean ± SD	19.9 ± 10.3	19.7 ± 9.5	20.4 ± 11.8	0.880
	25%	13.7	13.8	10.2	
	75%	24.2	23.9	26.9	
N1 and N2 (%/TiB)	Mean ± SD	51.2 ± 12.2	50.0 ± 3.7	53.7 ± 8.8	0.380
	25%	43.6	40.2	45.5	
	75%	62.5	62.6	62.0	
N3 (%/TiB)	Mean ± SD	10.4 ± 6.4	10.6 ± 6.1	10.3 ± 7.0	0.706
	25%	5.4	5.7	5.1	
	75%	15.4	15.3	15.7	
REM (%/TiB)	Mean ± SD	14.3 ± 7.4	13.6 ± 7.3	15.5 ± 7.7	0.266
	25%	10.6	10.4	11.6	
	75%	17.5	17.0	20.4	
Sleep efficacy (%/TiB)	Mean ± SD	76.3 ± 12.3	74.9 ± 13.3	79.1 ± 10.3	0.300
	25%	68.7	63.6	75.6	
	75%	86.5	85.6	86.7	
WASO (minutes)	Mean ± SD	83.8 ± 57.3	89.6 ± 62.5	76.44 ± 44.7	0.686
	25%	43.0	37.5	44.0	
	75%	120.0	132.8	104.0	
Sleep onset latency (minutes)	Mean ± SD	31.9 ± 35.7	36.2 ± 40.5	20.6 ± 15.6	0.057
	25%	12.5	15.5	9.0	
	75%	41.0	43.5	32.0	
Sleep stage changes (*n*)	Mean ± SD	145.7 ± 47.4	148.8 ± 48.0	140.7 ± 47.8	0.493
	25%	119.0	120.5	106.0	
	75%	171.8	178.3	170.0	
Awakenings (*n*)	Mean ± SD	26.7 ± 12.4	27.8 ± 11.4	25.4 ± 13.8	0.187
	25%	18.0	20.5	18.0	
	75%	32.0	33.0	31.0	
PLM-index (*n*/h)	Mean ± SD	23.0 ± 29.7	19.8 ± 24.2	27.2 ± 37.2	0.757
	25%	2.3	2.9	1.8	
	75%	35.7	32.8	38.8	
PLM-arousal-index (*n*/h)	Mean ± SD	2.8 ± 4.2	2.5 ± 4.2	3.1 ± 4.3	0.695
	25%	0.2	0.2	0.2	
	75%	3.6	3.1	5.8	

OSA: obstructive sleep apnea; PLMD: periodic limb movement disorder; RLS: restless legs syndrome; VAS: Visual Analog Scale; MFIS: Modified Fatigue Impact Scale; PSQI: Pittsburgh Sleep Quality Index; ESS: Epworth Sleepiness Scale; BDI: Beck Depression Inventory; FSS: Fatigue Severity Scale; TiB: time in bed; N1: sleep stage NREM 1; N2: sleep stage NREM 2; N3: sleep stage NREM 3; REM: rapid eye movement; WASO: wake after sleep onset; PLM: periodic leg movement.
